# Risks of treated anxiety, depression, and insomnia among nurses: A nationwide longitudinal cohort study

**DOI:** 10.1371/journal.pone.0204224

**Published:** 2018-09-25

**Authors:** Charles Lung-Cheng Huang, Ming-Ping Wu, Chung-Han Ho, Jhi-Joung Wang

**Affiliations:** 1 Department of Psychiatry, Chi Mei Medical Center, Tainan, Taiwan; 2 Department of Social Work, Chia Nan University of Pharmacy and Science, Tainan, Taiwan; 3 Division of Urogynecology and Pelvic Floor Reconstruction, Department of Obstetrics and Gynecology, Chi Mei Medical Center, Tainan, Taiwan; 4 Center of General Education, Chia Nan University of Pharmacy and Science, Tainan, Taiwan; 5 Department of Medical Research, Chi Mei Medical Center, Tainan, Taiwan; 6 Department of Hospital and Health Care Administration, Chia Nan University of Pharmacy and Science, Tainan, Taiwan; Mohammed bin Rashid University of Medicine and Health Sciences, UNITED ARAB EMIRATES

## Abstract

The high level of occupational stress and burnout among nurses can lead to insomnia, anxiety, and depression. However, the actual risks for healthcare-seeking for these stress-related mental health problems among nurses are still unclear. The aim of this study was to explore the risks and influencing factors of treated anxiety, depression, and insomnia among nurses. We used claims data obtained from the 2010 National Health Insurance Research Database (NHIRD) in Taiwan. Hospital nurses who had at least 3 coded ambulatory care claims or 1 inpatient claim with a principal diagnosis of anxiety, depression, or insomnia were identified. A cohort of 46,120 nurses and 92,240 matched controls were included. All the study subjects were followed up until the onset of any of the aforementioned outcomes, death, or the end of 2012. Results showed that the adjusted hazard ratios (HRs) for treated anxiety, depression, and insomnia among all the nurses were 0.91 (95% CI, 0.88–0.95), 0.59 (95% CI, 0.55–0.63), and 1.43 (95% CI, 1.38–1.48), respectively. Furthermore, the risks of these psychiatric problems in healthcare-seeking nurses were affected by age, gender, hospital level, and job tenure. Our findings suggest that hospital nurses have lower hazards of treated anxiety and depression than the general population, although they have a higher hazard of treated insomnia. There may be undertreatment in some subgroups of nurses with different demographic and working characteristics.

## Introduction

Nursing has been acknowledged to be a stressful occupation with a high prevalence of distress and stress-related burnout [1–3]. The high level of occupational stress and burnout among nurses can lead to behavioral health problems and psychiatric morbidity, including insomnia [4–6], anxiety [7–10], depression [7, 10–14], and substance use [15–17].

In Taiwan, the working conditions of nurses have become exhausting and highly stressful in recent years due to heavy workloads and litigation, extended working hours and high levels of time-related pressure, a lack of control over work, and tense nurse–patient relationships. Consequently, increasing numbers of nurses in Taiwan feel frustrated and burned out in their jobs, and related behavioral health issues have drawn public attention. For example, a recent Taiwanese study reported that the patterns of occasional, frequent, and daily benzodiazepine (BZD) use among nurses exhibited an increasing trend [15]. Moreover, the risk of frequent BZD use (as opposed to infrequent BZD use) increased significantly when nurses exhibited comorbid depression, anxiety, or sleep disorders.

Distress and stress-related behavioral health problems among nurses are turning into a worldwide public health issue. When health providers are not well, it may lead to declines in the quality and quantity of the care they provide to patients [18–19]. A growing body of literature suggests that nurses have higher risks of anxiety, depression, and sleep disorders that require medical treatment [6–7, 10, 16–17, 20]. However, studies have also suggested that nurses appear to be resistant to seeking care for their psychological or behavioral health problems due to concerns about confidentiality and stigma [17, 21–22].

There have only been limited studies thus far on the healthcare-seeking behavior for stress-related psychiatric problems among nurses [4, 15], and the actual risks for treated anxiety, depression, and insomnia among nurses are still unclear. One recent study investigated the incidence of treated insomnia in female nursing staff using a nationwide representative sample in Taiwan [4], and found that female nurses had higher incidences of adjustment insomnia and psychophysiological insomnia but had a lower incidence of nonorganic insomnia than did other female medical personnel. To fill the gaps in the existing literature, the object of this study was to explore the risks and influencing factors of treated anxiety, depression, and insomnia among nurses. Using a nationwide population-based database containing data on registrants in Taiwan’s National Health Insurance program (NHIRD), our goal was to examine the hypothesis that healthcare-seeking nurses may have higher hazards of treated anxiety, depression, and insomnia due to having higher risks of these psychiatric problems than the general population.

## Material and methods

### Data source

Taiwan’s National Health Insurance Research Database (NHIRD) was used in this population-based cohort study. The NHIRD covers about 99% of the population in Taiwan and identifies the diagnoses given to the individual patients included within it according to the International Classification of Diseases, Ninth Revision, Clinical Modification (ICD-9-CM). This claims database also includes the baseline information of registered medical personnel, such as working status, licensure date, and working hospital level. Using encrypted identification numbers, the clinical visits of those medical personnel can be identified among the NHIRD data in order to analyze their medical records.

The controls were defined as subjects who were not medical personnel. The Longitudinal Health Insurance Database 2000 (LHID2000), which contains 1 million randomly selected patients excluding medical personnel from the year 2000 Registry of Beneficiaries of the NHIRD, was used in this study to identify the controls from the general population.

### Selection and definition of study subjects

**Fig 1:**
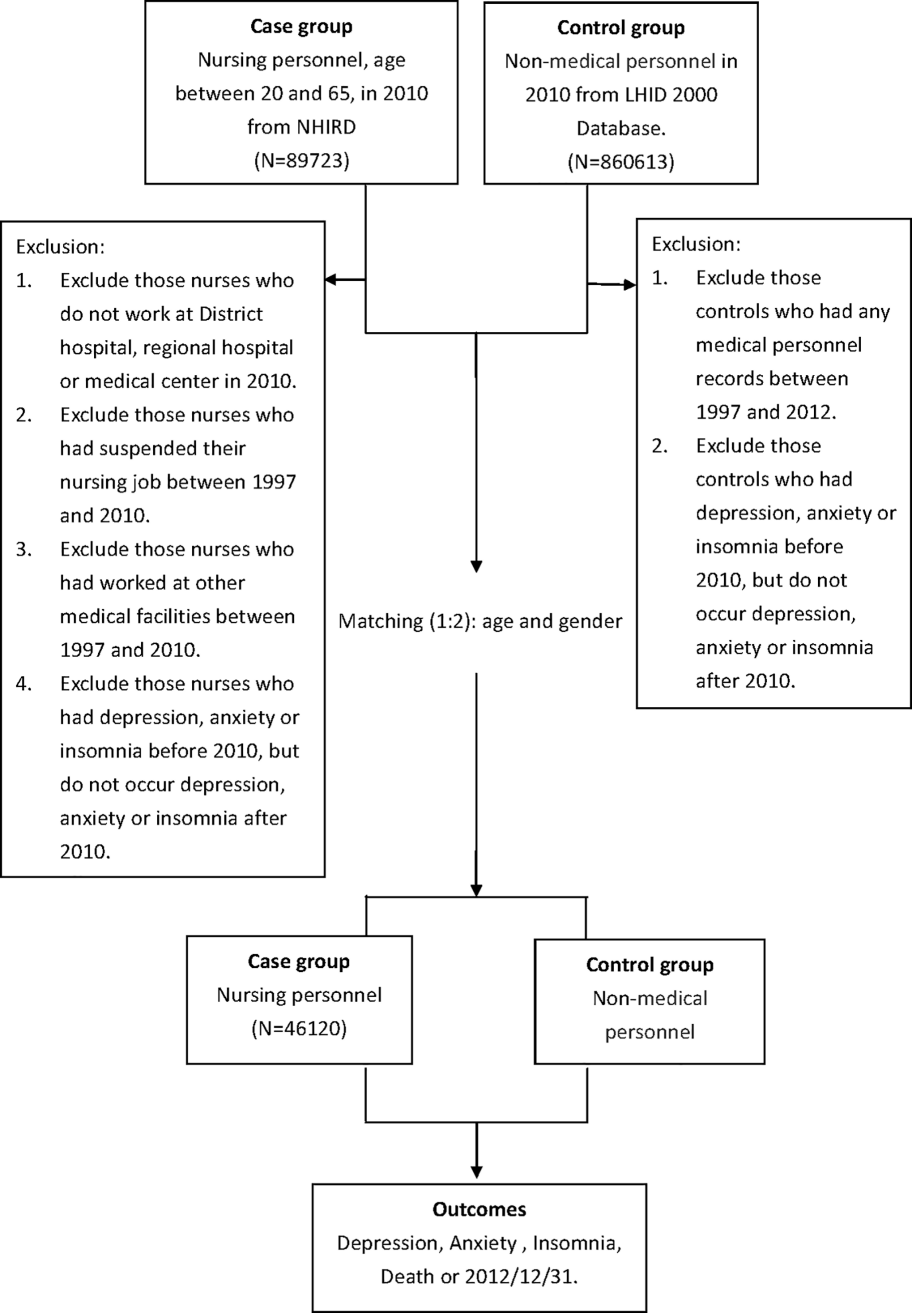
Flowchart showing the selection of the patient populations evaluated in the study. ^a^LHID = Longitudinal Health Insurance Database 2000.

The registered nurses aged 20 to 65 years old in the 2010 medical personnel database from NHIRD were selected in this study. In order to provide estimates for the investigated group under a similar standard, those registered nurses who ever worked below the level of a local hospital or who interrupted their nursing job before 2010 were excluded. Nurses who work in clinics were excluded because the working environment and demands of clinics and hospitals are quite different. The comparison cohort consisted of the general population excluding medical personnel from the LHID2000. The controls had the same criteria as nurses. Subjects from the general population were selected from LHID2000 in 2010, and those with history of anxiety, depression, and insomnia before 2010 were also excluded. The comparison cohort was also matched according to age and gender at a 2:1 ratio. All the study subjects were followed up until the onset of new outcomes (anxiety, depression, and insomnia), death, or December 31, 2012. Fig 1 presents a flowchart of the study subject selection process.

In addition, potential confounders, including age, gender, working hospital level, job tenure, Charlson comorbidity index (CCI) score, and major medical comorbidities, were also considered in this study. The working hospital levels of the individuals in the nurses group were defined according to where they were working as of 2010, with the different workplaces categorized as medical center, regional hospital, or local hospital. The job tenure for each nurse was defined as the number of years worked by the given nurse from 1997 to 2010.The comorbidities of diabetes mellitus (DM, ICD-9-CM: 250), hypertension (HTN, ICD-9CM codes: 401–405), coronary artery disease, (CAD, ICD-9CM code: 410–414), and hyperlipidemia (ICD-9-CM codes: 272), as well as CCI scores, were counted if they were noted for >3 ambulatory care claims or 1 inpatient claim coded before 2010.

### Measurements

The outcomes of interest in this study included anxiety, depression, and insomnia. All of the study subjects were followed from 2010 until the end of 2012 to determine the risks of anxiety, depression, and insomnia during that period. A subject was viewed as having anxiety on the basis of his or her records indicating any of the following diagnoses (the ICD-9CM code for each of which is shown in the associated parentheses): anxiety states (300.0;included 300.00 Anxiety state, 300.01 Panic disorder without agoraphobia, 300.02 Generalized anxiety disorder, and 300.09 Other anxiety states); phobic disorders (300.2); obsessive-compulsive disorders (300.3); and adjustment disorder with anxiety (309.24). A subject was viewed as having depression on the basis of his or her records indicating any of the following diagnoses (the ICD-9CM code for each of which is shown in the associated parentheses): major depressive disorder, single episode (296.2); major depressive disorder, recurrent episode (296.3); bipolar I disorder, most recent episode (or current) of depression (296.5); atypical depressive disorder (296.82); depressive disorder, not elsewhere classified (311); dysthymic disorder (300.4); adjustment disorder with depressed mood (309.0); and prolonged depressive reaction (309.1). A subject was viewed as having insomnia on the basis of his or her records indicating any of the following diagnoses (the ICD-9CM code for each of which is shown in the associated parentheses): transient disorder of initiating or maintaining sleep (307.41); persistent disorder of initiating or maintaining sleep (307.42); and unspecified insomnia (780.52). More specifically, a subject was viewed as having any of the above outcomes (that is, anxiety, depression, or insomnia) if at least one of the associated diagnoses was identified for at least 3 outpatient service claims in 1 year or if the subject had at least 1 inpatient hospitalization claim for the outcome during the full study period. We set such criterion in order to increase diagnostic validation, which has been used in previous similar studies [23–24].

### Statistical analyses

The categorical variables are reported as frequency with percentage, and the continuous variables are presented as median with interquartile range (IQR). For the comparison of baseline characteristics and comorbidities between the nurses and the general population, Pearson’s chi-square test for categorical variables and Wilcoxon’s rank sum test for continuous variables were used. The Cox regression model was used to estimate the risks of new-onset anxiety, depression, and insomnia for the nurses and the general population, respectively, with adjustments for age, gender, comorbidities, and CCI score. Stratified analyses in terms of age groups, gender, comorbidities, CCI scores, working hospital level, and job tenure were also performed. In order to further compare the risks of anxiety, depression, and insomnia among nurses with different characteristics, the Cox regression model was used to measure the potential risk factors for new-onset anxiety, depression, and insomnia among various subgroups of nurses. In addition, Kaplan-Meier curves were used to describe the trend among nurses who remained risk-free of new-onset anxiety, depression, or insomnia with log-rank test to compare the risk difference between job tenure. SAS 9.4 (SAS Institute, Cary, NC, USA) was used for all analyses, and the statistical significance level was set at 0.05 (two-tails).

### Ethics statement

According to regulations of the Bureau of National Health Insurance in Taiwan, personal identifiers are encrypted in the NHIRD to protect patients’ privacy and prevent the possibility of an ethical violation. For the purpose of research, the anonymous identification numbers have linked claims information, such as gender, month of birth, medical services received, and prescription information. Therefore, informed consent was not required for the present study, which was approved for exemption by the Institutional Review Board (IRB) of Chi Mei Medical Center (IRB: 10206-E06).The study was carried out in accordance with The Code of Ethics of the World Medical Association (Declaration of Helsinki).

## Results

### Sample characteristics

A total of 46,120 nurses and 92,240 matched controls were enrolled. There were no significant differences in age, gender, or the prevalences of DM and CAD between the two groups, although the prevalences of HTN and hyperlipidemia, as well as the mean CCI score, were higher among the nurses (Table 1). Furthermore, compared to the controls, the nurses had a significantly lower rate of depression but a higher rate of insomnia during 3-year follow-up.

**Table 1:** Comparison of demographic and clinical characteristics of nurses and general population.

	General population (%) (N = 92240)	Nurses (%) (N = 46120)	p-value
Age group, n(%)			
< = 29	42198(45.75)	21099(45.75)	1.0000
30–44	42480(46.05)	21240(46.05)	
> = 45	7562(8.20)	3781(8.20)	
Gender, n(%)			
Male	1730(1.88)	865(1.88)	1.0000
Female	90510(98.12)	45255(98.12)	
Comorbidities, n(%)			
DM	1000(1.08)	505(1.09)	0.8546
HTN	2001(2.17)	1212(2.63)	< .0001
CAD	264(0.29)	148(0.32)	0.2642
Hyperlipidemia	1344(1.46)	1455(3.15)	< .0001
CCI score, n(%)			
0	85611(92.81)	40305(87.39)	< .0001
1	5071(5.50)	4625(10.03)	
> = 2	1558(1.69)	1190(2.58)	
Hospital level, n(%)			
Medical center	n/a	18499(40.09)	
Regional hospital	n/a	19776(42.88)	
Local hospital	n/a	7855(17.03)	
Job tenure (years), n(%)			
< = 3	n/a	19607(42.52)	
4–8	n/a	17786(38.56)	
> = 9	n/a	8727(18.92)	
Outcome			
Anxiety, n(%)	8828(9.57)	4382(9.50)	0.6789
Time to Anxiety(months), median (IQR)	14.70(4.84–24.38)	14.05(4.61–23.95)	0.1691
Depression, n(%)	3597(3.90)	1159(2.51)	< .0001
Time to Depression(months), median (IQR)	12.70(3.39–22.83)	12.86(2.99–22.93)	0.8606
Insomnia, n(%)	7845(8.50)	5864(12.71)	< .0001
Time to Insomnia(months), median (IQR)	14.31(5.03–24.34)	13.45(4.34–23.65)	0.0039

Note: The p-values are from the Pearson’s chi-squared test for categorical variables and from Wilcoxon’s rank-sum test for continuous variables.

### Nurses vs. general population

After controlling for medical comorbidities including DM, HTN, CAD, and hyperlipidemia, as well as for CCI score, the adjusted hazard ratio (HR) for anxiety among all nurses was 0.91 (Table 2). Nurses in the< = 29 years old age group had a significantly higher HR for anxiety (1.17) than the general population, while nurses in the 30–44 years old age group and > = 45 years old age group had lower HRs for anxiety (0.85 and 0.63, respectively) than the general population. Female nurses had a significantly lower HR for anxiety (0.91) than controls. Compared to the general population, nurses working in medical centers also had a significantly lower HR for anxiety (0.64), while nurses working in local hospitals had a higher HR for anxiety (1.42). Nurses with job tenures of< = 3 and > = 9 years also had lower HRs for anxiety (0.80 and 0.93, respectively) than controls.

**Table 2:** Overall and stratified hazard ratios of anxiety, depression, and insomnia among nurses (vs. controls).

	Anxiety	Depression	Insomnia
	Adjusted HR (95% C.I.)	Adjusted HR (95% C.I.)	Adjusted HR (95% C.I.)
Overall	0.91(0.88–0.95)**	0.59(0.55–0.63)**	1.43(1.38–1.48)**
Age group			
< = 29	1.17(1.10–1.24)**	0.66(0.59–0.73)**	1.95(1.84–2.06)**
30–44	0.85(0.81–0.90)**	0.57(0.52–0.63)**	1.33(1.26–1.39)**
> = 45	0.63(0.57–0.69)**	0.46(0.37–0.57)**	0.79(0.72–0.88)**
Gender			
Male	1.08(0.76–1.53)	0.63(0.33–1.18)	2.18(1.63–2.91)**
Female	0.91(0.88–0.95)**	0.59(0.55–0.63)**	1.42(1.37–1.47)**
Hospital level			
Medical center	0.64(0.60–0.68)**	0.45(0.41–0.50)**	1.12(1.06–1.18)**
Regional hospital	0.97(0.92–1.02)	0.60(0.55–0.66)**	1.44(1.38–1.51)**
Local hospital	1.42(1.33–1.51)**	0.85(0.76–0.96)*	2.09(1.97–2.21)**
Job tenure			
< = 3	0.80(0.76–0.84)**	0.54(0.49–0.59)**	1.25(1.19–1.31)**
4–8	1.03(0.98–1.09)	0.66(0.60–0.72)**	1.65(1.58–1.73)**
> = 9	0.93(0.87–0.99)*	0.57(0.50–0.65)**	1.30(1.27–1.44)**

*p<0.05.

**p < .0001.

Note: The model was compared with the general population, and the risk was adjusted for age, gender, medical comorbidities, and CCI score.

After controlling for medical comorbidities including DM, HTN, CAD, and hyperlipidemia, as well as for CCI score, the adjusted HR for depression among all nurses was 0.59 (Table 2). Nurses in all three age groups (i.e., < = 29 years old, 30–44 years old, and > = 45 years old) had significantly lower HRs for depression (0.66, 0.57, and 0.46, respectively) than the general population. Female nurses also had a significantly lower HR for depression (0.59), as did nurses working at all three hospital levels (i.e., medical centers, regional hospitals, and local hospitals; 0.45, 0.60, and 0.85, respectively). Nurses in all three job tenure groups (i.e.,< = 3, 4–8, and > = 9 years)likewise had significantly lower HRs for depression(0.54, 0.66, and 0.57, respectively).

After controlling for medical comorbidities including DM, HTN, CAD, and hyperlipidemia, as well as for CCI score, the adjusted HR for insomnia among all nurses was 1.43 (Table 2).Nurses in the< = 29 years old age group and 30–44 years old age group had significantly higher HRs for insomnia (1.95 and 1.33, respectively) than the general population, while nurses in the> = 45 years old age group had a lower HR for insomnia (0.79).Both male and female nurses had significantly higher HRs for insomnia(2.18 and 1.42, respectively), as did nurses working at all three hospital levels (i.e., medical centers, regional hospitals, and local hospitals;1.22, 1.44, and 2.09, respectively). Nurses in all three job tenure groups (i.e., < = 3 years, 4–8 years, and > = 9 years) also had significantly higher HRs for insomnia (1.25, 1.65, and 1.30, respectively).

### Comparison among nurses

Compared to the < = 29 years old age group, nurses in the 30–44 years old age group had significantly higher HRs for anxiety, depression, and insomnia (1.13, 1.17, and 1.14, respectively)(Table 3), while nurses in the > = 45 years old age group had higher HRs for anxiety and insomnia (1.39 and 1.17, respectively).

**Table 3:** Comparison of hazard ratios of anxiety, depression, and insomnia among nurses.

	Anxiety	Depression	Insomnia
	Adjusted HR (95% C.I.)	Adjusted HR (95% C.I.)	Adjusted HR (95% C.I.)
Age group			
< = 29	1.00(ref.)	1.00(ref.)	1.00(ref.)
30–44	1.13(1.05–1.21)*	1.17(1.03–1.34)*	1.14(1.07–1.12)**
> = 45	1.39(1.25–1.55)**	1.11(0.89–1.38)	1.17(1.06–1.29)*
Gender			
Male	0.61(0.46–0.81)*	0.59(0.34–1.03)	0.92(0.76–1.12)
Female	1.00(ref.)	1.00(ref.)	1.00(ref.)
Hospital level			
Medical center	1.00(ref.)	1.00(ref.)	1.00(ref.)
Regional hospital	1.45(1.35–1.56)**	1.31(1.14–1.50)*	1.22(1.15–1.29)**
Local hospital	2.17(2.00–2.36)**	1.89(1.61–2.21)**	1.81(1.69–1.94)**
Job tenure			
< = 3	1.00(ref.)	1.00(ref.)	1.00(ref.)
4–8	1.09(1.02–1.17)*	1.11(0.97–1.27)	1.15(1.09–1.22)**
> = 9	1.13(1.04–1.23)*	1.00(0.85–1.18)	1.10(1.02–1.18)*

*p<0.05.

**p < .0001.

Note: The model was adjusted for the medical comorbidities and CCI score.

Male nurses had a significantly lower HR (0.61) for anxiety when compared to female nurses (Table 3).

Compared to the medical center group, nurses working in regional hospitals had significantly higher HRs for anxiety, depression, and insomnia (1.45, 1.31, and 1.22, respectively) (Table 3), while nurses working in local hospitals had significantly higher HRs for anxiety, depression, and insomnia (2.17, 1.89, and 1.81, respectively).

Compared to the nurses with job tenures of< = 3 years, nurses with job tenures of 4–8 years had significantly higher HRs for anxiety and insomnia (1.09 and 1.15, respectively) (Table 3), while nurses with job tenures of> = 9 years had significantly higher HRs for anxiety and insomnia (1.13 and 1.10, respectively).

**Fig 2:**
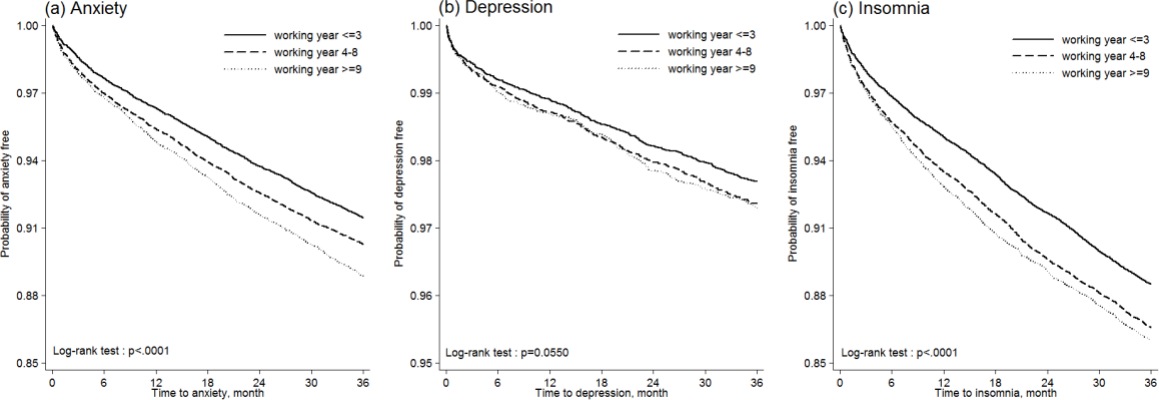
Kaplan-Meier curves of anxiety, depression, and insomnia risk by job tenure.

The Kaplan-Meier plots showed that nurses with longer job tenures had significantly higher risks of developing anxiety and insomnia (Fig 2).

## Discussion

### Anxiety, depression, and insomnia among healthcare-seeking nurses

This study aimed to explore the actual risks for treated stress-related psychiatric problems in nurses. Using a large sample from a nationally representative database, we designed a 3-year cohort study to examine the hazard ratios of treated anxiety, depression, and insomnia among hospital nurses.

Generally, based on hazard ratios of 3-year data, the results of the current study suggest that nurses have lower hazards of treated anxiety and depression than the general population, although they have a higher hazard of treated insomnia. This latter result is partially consistent with that of a previously reported study, which found that healthcare-seeking nurses were at higher risks for some subtypes of insomnia[4]. Due to the abundant evidence of higher prevalences of depression and anxiety among nurses than among the general population[7–14], the apparent undertreatment of these problems among the nurses in the present study may be due to resistance or other barriers to help-seeking.

### The impact of age, gender, and working characteristics

The risks of these psychiatric problems in healthcare-seeking nurses seem to vary among different demographic subgroups. Our data showed, for example, that middle aged and late middle aged nurses had significantly higher HRs for anxiety, depression, and insomnia when compared to younger nurses, although the middle aged and late middle aged nurses still had significantly lower risks for anxiety, depression, and insomnia than the general population. This finding was in line with those of other studies, which reported that age intensified incidences of anxiety as well as abnormal parasympathetic activity [25] and that sleep problems due to shift work disorder were associated with increased age [26].

As for gender, male nurses had a significantly lower HR for anxiety when compared to female nurses, although female nurses still had a significantly lower risk for anxiety than the general population. Although existing literature suggest that there were gender-specific prevalences and symptoms profiles in depression and anxiety among the general population [27–29], there has previously been little research concerning gender differences in the manifestation of stress-related psychiatric problems among health-seeking nurses. However, one recent study in South Korea found that gender was related to insomnia and functional dyspepsia in shift-working nurses [30]. The risk of psychiatric problems in healthcare-seeking nurses may also be affected by working characteristics, such as hospital level and job tenure. Compared to the medical center group, nurses working in regional hospitals and local hospitals had higher HRs for anxiety, depression, and insomnia. The distinct workloads and stressors among different hospital levels may be the reason for this finding. Another possible explanation is that there may be discrepancies in the accessibility of help and barriers to help-seeking among different hospital levels.

It is interesting that job tenure appears to have an impact on these stress-related psychiatric problems. Among the healthcare-seeking nurses in this study, those with longer job tenures had significantly higher risks of developing anxiety and insomnia. This finding echoes those of reports indicating that middle and late career nurses show higher sensitivity to anxiety than early career nurses [8] and that greater years of service aggravate the risks of anxiety and abnormal parasympathetic activity [25]. Working tenure has also been found to be a significant predictor of stress levels, depression, and intention to leave in nurses [14].

### Undertreatment of anxiety and depression among nurses

Even though past studies suggest that nurses may be more vulnerable to stress-related psychiatric disorders, our study discovered that nurses do not seem to seek medical help for certain types of disorders, especially for anxiety and depression. The reasons why nurses are resistant to seeking help for psychological or behavioral health problems may be due, in part, to the nurses’ own attitudes regarding such problems. More specifically, the barriers appear to be that seeking help for mental health issues could be stigmatizing in terms of what peers and employers might think of them, which could, in turn, cause unfavorable consequences for their career development [17, 21]. Moreover, it is worth mentioning that such negative attitudes toward help seeking may emerge as early as when the nurses are students[22, 31].Generally speaking, there seems to be insufficient heed given to mental health problems and hesitation to seek help among student nurses due to concerns about confidentiality or for other reasons. However, there are still other possible explanations for the lower level of treated anxiety and depression than anticipatory estimates from community data, such as cultural factor. Some studies have revealed that Asian populations tend to respond to excessive stress with somatic symptoms rather than depressive or affective symptoms as in Western populations [32–33]. Some authors argued that Asian and Western differences in affective symptoms and somatization reflect discrepancies in help-seeking behavior instead of actual prevalence differences, because somatic complaints seen as more probably than affective symptoms to obtain treatment in developing countries[34–35].In other word, the differential reporting rates between affective and somatization symptoms in community and clinic-referred individuals who are seeking help for mental health problems were more prominent in Asian countries.

### Study limitations

The current study has some limitations. First, our sample consisted of hospital nurses only. Thus, discretion is needed when generalizing our findings to other types of nurses. Second, the anxiety, depression, and insomnia diagnoses used in the study were from administrative claims data reported by physicians and based on the ICD-9-CM,which may not be as accurate as diagnoses made by structured interview. To improve the diagnostic accuracy, we recruited only those subjects with anxiety, depression, and insomnia who had at least three outpatient service claims within one year or at least one inpatient hospitalization claim during the entire study period. Third, our study did not incorporate substance use disorder, overload, or workplace stressing conditions, which are other significant factors affecting nurses’ mental health. Finally, the possible reasons why nurses resistant or due to other barriers to help-seeking are purely speculative because there is no specific data to support these claims in the current study.

### Clinical implications

Notwithstanding these considerations, the present study still has valuable implications for research and practice. To the best of our knowledge, our study is among the few to simultaneously explore the risks of treated anxiety, depression, and insomnia among nurses using a nationwide population-based database with a longitudinal design. This study contributes to the existing body of knowledge regarding the influences of age, gender, and working characteristics on treated stress-related psychiatric disorders among nurses. The results of the study provide preliminary data that can be used by government health administrators, healthcare employers, and schools of nursing to promote early recognition of nurses' mental illness treatment needs.

## Conclusions

Hospital nurses have lower hazards of treated anxiety and depression than the general population, although they have a higher hazard of treated insomnia.

The risks of these psychiatric problems in healthcare-seeking nurses could be influenced by age, gender, hospital level, and job tenure. Our study discovered that even though nurses in certain subgroups may be more vulnerable to these stress-related psychiatric disorders, they do not seem to seek medical help for some of them. In other words, there may be undertreatment in some subgroups of nurses with different demographic and working characteristics. Findings from this research suggest that vital approaches may be needed to diminish the stigmatizing attitudes toward mental disorders among nurses, and to improve their prevention, identification, and treatment in the healthcare settings in which nurses practice. The study data also imply a need for more research to identify other factors affecting nurses’ wellness (such as substance use problems), how to reduce distress and stress-related burnout among nurses, how to enhance the early detection of behavioral health problems in this population, and how to remove barriers to their use of mental healthcare resources.

## Supporting information

S1 Supporting Information (DOCX)
