# Supplementary material for: Risks of treated anxiety, depression, and insomnia among nurses: A nationwide longitudinal cohort study
Source: PLoS One. 2018 Sep 25;13(9):e0204224. doi: 10.1371/journal.pone.0204224 (PMC6155527; doi:10.1371/journal.pone.0204224)
Supplement: S1 Supporting Information — (DOCX) [file pone.0204224.s001.docx]

Interested researchers can obtain the data through formal application to the HWDC, Department of Statistics, Ministry of Health and Welfare, Taiwan (<http://dep.mohw.gov.tw/DOS/np-2497-113.html>). All applications are reviewed for approval of data release and applicants must follow the Computer-Processed Personal Data Protection Law (<http://www.winklerpartners.com/?p=987>) and related regulations of National Health Insurance Administration.
